# Association of socioeconomic disadvantage and ethnicity with perinatal neonatal, and infant mortality in Slovakia

**DOI:** 10.1186/s12889-024-18645-0

**Published:** 2024-04-24

**Authors:** Lucia Bosakova, Katarina Rosicova, Daniela Filakovska Bobakova

**Affiliations:** 1 Department of Health Psychology and Research Methodology, Faculty of Medicine, P.J. Safarik University, Tr. SNP 1, Kosice, 040 01 Slovak Republic; 2 Olomouc University Social Health Institute (OUSHI), Palacky University in Olomouc, Univerzitni 22, 771 11 Olomouc, Czech Republic; 3 Department of Regional Development, Land-Use Planning and Environment, Kosice Self-Governing Region, Nam. Maratonu Mieru 1, 042 66 Kosice, Slovakia

**Keywords:** Perinatal mortality, Neonatal mortality, Infant mortality, Socioeconomic disadvantage, Ethnicity, Roma

## Abstract

**Background:**

Infant mortality rates are reliable indices of the child and general population health status and health care delivery. The most critical factors affecting infant mortality are socioeconomic status and ethnicity. The aim of this study was to assess the association between socioeconomic disadvantage, ethnicity, and perinatal, neonatal, and infant mortality in Slovakia before and during the COVID-19 pandemic.

**Methods:**

The associations between socioeconomic disadvantage (educational level, long-term unemployment rate), ethnicity (the proportion of the Roma population) and mortality (perinatal, neonatal, and infant) in the period 2017–2022 were explored, using linear regression models.

**Results:**

The higher proportion of people with only elementary education and long-term unemployed, as well as the higher proportion of the Roma population, increases mortality rates. The proportion of the Roma population had the most significant impact on mortality in the selected period between 2017 and 2022, especially during the COVID-19 pandemic (2020–2022).

**Conclusions:**

Life in segregated Roma settlements is connected with the accumulation of socioeconomic disadvantage. Persistent inequities between Roma and the majority population in Slovakia exposed by mortality rates in children point to the vulnerabilities and exposures which should be adequately addressed by health and social policies.

## Introduction

Perinatal, neonatal and infant mortality rates are reliable indices of the child and general population health status as well as health care delivery in a country because the health and survival of infants depend upon the characteristics of the society in which they are born [1, 2]. Preterm birth, complications during and immediately after childbirth, congenital disabilities, and infections are the leading causes of death in the most vulnerable period for child survival during the first month of life [3]. Later on, in the post-natal period, the leading causes of death are pneumonia, diarrhoea, and congenital disabilities [3].

Among the most critical factors affecting infant mortality are socioeconomic status and ethnicity. Previous European research confirmed that socioeconomic inequalities in health can already be seen at the very start of life, as shown by socioeconomic differences in stillbirths, perinatal, neonatal and infant mortality [4]. Ethnic disparities in infant mortality in Europe have been also reported [5–8].

Slovakia belongs to the countries with the highest infant mortality rates in the EU [9]. Generally, infant mortality has decreased in most European countries over the past ten years. Slovakia, however, has remained at the same level (4.9 deaths per 1,000 live births), above the EU average [9]. The most significant and most disadvantaged ethnic minority in Europe, with shorter life expectancy and high mortality rates, is Roma [10]. Approximately half of 440,000 Roma living in Slovakia reside in more or less segregated neighbourhoods and marginalised Roma communities [11].

Health inequalities between Roma and the majority of population are well documented across European countries with a significant Roma population, including Slovakia [12, 13]. The health of Roma living in social exclusion is endangered by numerous factors such as unsanitary dwellings and poor living conditions, environmental hazards, inadequate nutrition, extreme poverty and deprivation [14, 15]. Exposures and vulnerabilities of Roma communities, together with limited access to health care, contribute to worse health outcomes compared to the majority of populations in countries of residence [13, 16, 17]. In Slovakia, between 2005 and 2009, the infant mortality rate was more than three times higher, and the neonatal mortality rate was 2.5 times higher in municipalities with a dominant representation of Roma compared to municipalities with a low or no representation of Roma [18]. Between the years 1992 – 2012, the risk of death of children under one year from Roma communities was approximately 2.5 times higher compared to the entire population of Slovakia [19]. The most recent data reporting period between 1996 and 2018 suggests that although mortality rates in the age group 0–14 are generally decreasing, the rates in the districts with the most significant Roma population in Slovakia are more than four times higher [20].

However, to our knowledge, the latest available data on differences in infant mortality by ethnicity and socioeconomic disadvantage is not well documented, yet. In addition, we assume that the pandemic period of COVID-19 could also have had a significant impact and even more endangered the already vulnerable position of Roma living in Slovakia. Therefore, this study assessed the association between socioeconomic disadvantage, ethnicity and perinatal, neonatal and infant mortality in Slovakia before and during the COVID-19 pandemic. We believe it could bring vital information for designing public health policy measures within Slovakia and throughout Europe concerning other socioeconomically disadvantaged and ethnic groups.

## Methods

We used an ecological design typical when looking for geographical correlations between mortality or disease incidence and the prevalence of risk factors whereas the unit of observation is the population of community [21].

### Study population

The study population covers all inhabitants of the Slovak Republic in the period 2017–2022. The study population was analysed at the district level to explore regional differences. The Slovak Republic is divided into 79 districts, five of which constitute the capital city, Bratislava and four of the second largest city, Košice.

### Data

The basic data consists of absolute population numbers and numbers of live births, stillbirths and deaths in the districts of the Slovak Republic in the period 2017–2022. This data reflects the monitoring of natural changes in the population of the Slovak in the period 2017–2022 and were obtained from the Statistical Office of the Slovak Republic. Population registration is meticulously documented for maximum accuracy. Health facilities are required to submit a birth report on the first working day following a child's birth and a death report within three working days after examining a deceased individual. Mortality rates were calculated for each district of the Slovak Republic. The perinatal mortality rate is expressed as the number of stillbirths and deaths under seven days of age per the number of births. Neonatal mortality rate is defined as the number of deaths of children aged 0–27 days per the number of live births. The infant mortality rate is expressed as the number of deaths under one year of age per the number of live births [22].

Educational level, long-term unemployment rate and the proportion of the Roma population were used as indicators of socioeconomic disadvantage influencing mortality rates. Indicators were computed individually for each district due to data availability and increased reliability at the district level. Educational level is expressed as the percentage of inhabitants over 15 years of age with only elementary or uncompleted elementary education; both were based on the 2021 population census from the Statistical Office of the Slovak Republic. The long-term unemployment rate was obtained from the tally of the Office of Labour, Social Affairs and Family of the Slovak Republic. The long-term unemployment rate (12 months and more) was expressed as the proportion of unemployed persons seeking a job for longer than one year to the total number of registered unemployed. The percentage of the Roma population living in settlements was obtained from Atlas of Roma Communities—the sociographic mapping conducted by the Office of the Government Plenipotentiary for the Roma Community in cooperation with the Institute for Work and Family Research undertaken in 2019. Atlas of Roma communities covers municipalities with a Roma settlement with more than 30 inhabitants or more than 30% share of Roma in the total population in case there is no Roma settlement in the municipality.

### Statistical analysis

As regards descriptive analyses, average mortality rates and mean proportion of the socioeconomic indicators were computed. After descriptive analyses, linear regressions were applied: perinatal, neonatal and infant mortality rates were used as the dependent variables in five separate regression models; the long-term unemployment rate, the educational level and the proportion of the Roma population were set as independent variables (factors). Firstly, the crude effects of each factor were explored separately, and then all factors were included in the final model. The analyses were repeated separately for the period preceding the COVID-19 pandemic (2017–2019) and encompassing the pandemic (2020–2022). The variance inflation factor (VIF) was used to detect and measure the amount of collinearity of independent variables in the final regression model. All data was analysed using SPSS, version 21. Maps were constructed using the regional mortality rates and data by socioeconomic indicators in ArcView. The range of the indicators on the maps was divided into equal intervals.

## Results

### Study population

The average number of inhabitants per year in the Slovak Republic from 2017–2022 was 5.45 million. The average number of births in 2017–2022 was 56,598, and the average number of live births in 2017–2022 was 56,424. The average number of stillbirths and deaths under seven days of age was 284. The average number of deaths of children aged 0–27 days, not including stillbirths, was 167. The average number of deaths under one year of age, not including stillbirths, was 282.

### Mortality

**Fig. 1:**
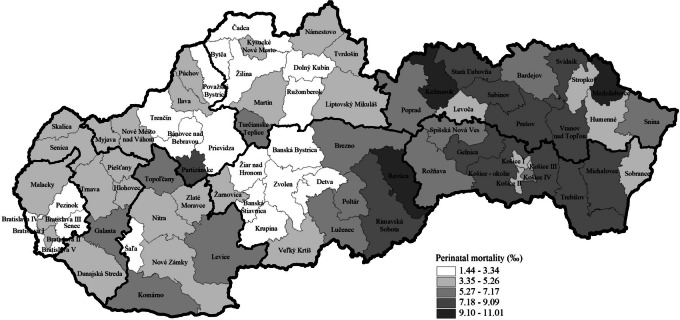
Perinatal mortality rate by districts in the Slovak Republic in the period 2017–2022 using data from the Statistical Office of the Slovak Republic

**Fig. 2:**
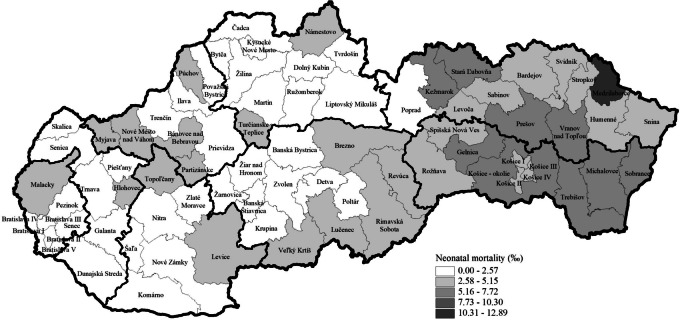
Neonatal mortality rate by districts in the Slovak Republic in the period 2017–2022 using data from the Statistical Office of the Slovak Republic

**Fig. 3:**
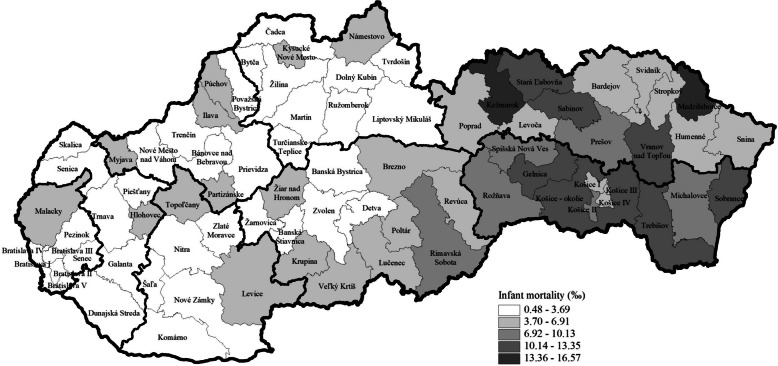
Infant mortality rate by districts in the Slovak Republic in the period 2017–2022 using data from the Statistical Office of the Slovak Republic

Regional differences in the perinatal, neonatal and infant mortality rates in the districts of the Slovak Republic in the period 2017–2022 are shown in Figs. 1, 2 and 3.

Figure 1 shows the perinatal mortality rate. Districts in Slovakia's northwestern and central parts are among those with the lowest perinatal mortality rate. Districts in the east part of Central Slovakia and districts of East Slovakia have a relatively high perinatal mortality rate. The majority of the districts (45 from 79) attained a lower perinatal mortality rate than the average national perinatal mortality rate, which was 5.02‰ from 2017–2022 (Fig. 1).

Figure 2 shows the neonatal mortality rate. Districts of West Slovakia and districts in the north part of Central Slovakia are among those with the lowest neonatal mortality rate. Districts in the eastern part of Slovakia are among those with the highest infant mortality rate. Compared with the neonatal mortality rate at the national level, which reached a value of 2.97‰ in the period 2017–2022, the majority of the districts (48 from 79) achieved a lower mortality rate than the average rate for the Slovak Republic (Fig. 2).

Figure 3 shows the infant mortality rate. Districts of the Slovak Republic can be divided into two specific regions by infant mortality. Districts of West Slovakia are among those with the lowest infant mortality rate. In contrast, districts in the eastern part of Slovakia have the highest infant mortality rate, especially in the central part of East Slovakia. Compared with the infant mortality rate at the national level, which reached a value of 5.00‰ in 2017–2022, the majority of the districts (51 from 79) achieved a lower mortality rate than the average rate for the Slovak Republic (Fig. 3).

### Education, unemployment and the Roma population

The basic data of perinatal, neonatal and infant mortality and selected socioeconomic indicators is shown in Table 1. The mean proportion of inhabitants with only elementary or uncompleted elementary education in the Slovak Republic was 16.97% (ranging from 8.06 to 29.15% per district). Long-term unemployment rate mean value in the Slovak Republic was 40.14% in 2017–2022 (ranging from 14.71 to 61.07%). The average percentage of Roma per district in the Slovak Republic was 7.63% (ranging from 0.00 to 33.12%).

**Table 1:** Basic data on perinatal, neonatal and infant mortality and socioeconomic indicators for the Slovak population in the period 2017–2022

	Slovakia total	District with a minimum value	District with maximum value
Perinatal mortality	5.02‰	1.44‰	11.01‰
Neonatal mortality	2.97‰	0.00‰	12.89‰
Infant mortality	5.00‰	0.48‰	16.57‰
Education elementary^a^	16.97%	8.06%	29.15%
Education uncompleted elementary^a^	0.28%	0.07%	0.75%
Long-term unemployment	40.14%	14.71%	61.07%
Proportion of Roma population^b^	7.63%	0.00%	33.12%

Source of data: Statistical Office of the Slovak Republic, Office of Labour, Social Affairs and Family of the Slovak Republic, Office of the Government Plenipotentiary for Roma Community, Institute for Work and Family Research

^a^Population census, 2021

^b^Sociographic mapping of Roma settlements, 2019

**Fig. 4:**
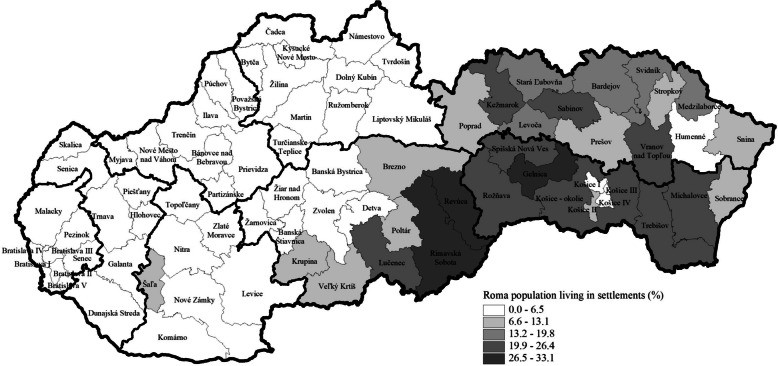
The proportion of the Roma population living in settlements by districts in the Slovak Republic in the year 2019 using data from the Statistical Office of the Slovak Republic, Office of the Government Plenipotentiary for Roma Community, Institute for Work and Family Research

Figure 4 shows the spatial distribution of the Roma population living in settlements by districts in 2019. The Slovak Republic is divided into two parts—districts in the western part of Slovakia are characterised by small proportions of the Roma population living in settlements, in contrast with districts in the eastern part of Slovakia, especially in the south and central part of East Slovakia, which are among those with high proportions of the Roma population living in settlements (Fig. 4).

Table 2 presents the results of the linear regression between the rates of perinatal, neonatal, and infant mortality in the districts of the Slovak Republic in the period 2017–2022 and the separate socioeconomic indicators. In this model, the variables were entered consecutively to explore the effects separately. Dependent variables are the rates of perinatal, neonatal and infant mortality individually. All the explored indicators of socioeconomic disadvantage showed a significant impact on perinatal, neonatal and infant mortality.

**Table 2:** Linear regression between perinatal, neonatal and infant mortality rate and socioeconomic indicators (separately) in the period 2017–2022

		Education – uncompleted elementary	Education – elementary	Education – uncompleted elementary and elementary	Long-term unemployment	Proportion of the Roma population
Perinatal mortality	Standardised Coefficients (Beta)	.696	.698	.702	.596	.732
	Sig	.000***	.000***	.000***	.000***	.000***
	R^2^	.485	.488	.493	.355	.537
Neonatal mortality	Standardised Coefficients (Beta)	.615	.462	.469	.540	.562
	Sig	.000***	.000***	.000***	.000***	.000***
	R^2^	.378	.214	.220	.291	.316
Infant mortality	Standardised Coefficients (Beta)	.675	.651	.655	.707	.759
	Sig	.000***	.000***	.000***	.000***	.000***
	R^2^	.455	.424	.430	.499	.576

Source of data: Statistical Office of the Slovak Republic, Office of Labour, Social Affairs and Family of the Slovak Republic, Office of the Government Plenipotentiary for Roma Community, Institute for Work and Family Research

^***^p ≤ 0.001 (2-tailed); R2 – explained variance

Table 3 presents the results of linear regression models between socioeconomic indicators in the districts of the Slovak Republic and the rates of perinatal, neonatal and infant mortality in the period 2017–2022 and separately in the periods 2017–2019 (the period preceding the COVID-19 pandemic) and 2020–2022 (the period encompassing the pandemic). The model explores the influence of all variables together on all three mortality rates. Individual models explain from 18.5% of the variance (neonatal mortality in the period 2017–2019) to 60.1% of the variance (infant mortality in the period 2017–2022). The educational level (proportion of inhabitants with uncompleted elementary or elementary education) and the proportion of the Roma population significantly affected perinatal mortality in 2017–2022. Long-term unemployment and the proportion of the Roma population contributed to the prediction of infant mortality in the districts of the Slovak Republic in the period 2017–2022. The effect of selected socioeconomic indicators on neonatal mortality in 2017–2022 was insignificant. In the period preceding the COVID-19 pandemic (2017–2019), the regression models show that the proportion of the Roma population significantly predicts perinatal mortality and long-term unemployment significantly predicts infant mortality. In the period encompassing the pandemic (2020–2022), only a proportion of the Roma population contributed to the prediction of neonatal and infant mortality in the districts of the Slovak Republic. The VIF used to detect the collinearity of independent variables in the final regression model showed that the proportion of the Roma population had a significant influence on the other variables, which may have reduced their resulting influence in the final regression model. Thus, excluding the proportion of the Roma population from the model would increase the significance of education and unemployment.

**Table 3:** Linear regression between perinatal, neonatal and infant mortality rate and socioeconomic indicators – all variables together – in the periods 2017 – 2022, 2017 – 2019 and 2020 – 2022

	**Perinatal mortality**		**Neonatal mortality**		**Infant mortality**	
	Standardised Coefficients (Beta)	Sig	Standardised Coefficients (Beta)	Sig	Standardised Coefficients (Beta)	Sig
**2017 – 2022**						
Education – uncompleted elementary and elementary	.299	.036*	-.011	.950	.060	.652
Long-term unemployment	-.008	.952	.243	.138	.262	.041*
Proportion of the Roma population	.489	.006*	.374	.086	.496	.004**
**R**^**2**^	**.563**		**.336**		**.601**	
**2017 – 2019**						
Education – uncompleted elementary and elementary	.260	.114	.115	.549	.164	.304
Long-term unemployment	-.021	.884	.284	.102	.370	.011*
Proportion of the Roma population	.423	.034*	.067	.772	.185	.337
**R**^**2**^	**.409**		**.185**		**.439**	
**2020 – 2022**						
Education – uncompleted elementary and elementary	.213	.226	-.204	.255	-.085	.557
Long-term unemployment	-.082	.624	-.091	.594	-.022	.875
Proportion of the Roma population	.437	.053	.767	.001**	.816	.000***
**R**^**2**^	**.316**		**.287**		**.530**	

Source of data: Statistical Office of the Slovak Republic, Office of Labour, Social Affairs and Family of the Slovak Republic, Office of the Government Plenipotentiary for Roma Community, Institute for Work and Family Research

^*^p ≤ 0.05

^**^p ≤ 0.01

^***^p ≤ 0.001 (2-tailed); R2 – explained variance

## Discussion

The results of our study point to the association of socioeconomic disadvantage and ethnicity with perinatal, neonatal and infant mortality. We found that on a regional level, each of the selected indicators of disadvantage (proportion of people with only elementary education, long-term unemployment, and proportion of Roma population) negatively affects perinatal, neonatal, and infant mortality rates. The proportion of the Roma population seems to have the most significant impact on mortality in the selected period between 2017 and 2022, especially during the COVID-19 pandemic (2020–2022).

All the explored indicators of socioeconomic disadvantage showed significant crude effects on perinatal, neonatal and infant mortality in Slovakia. The higher proportion of people with only elementary education and long-term unemployed, as well as the higher proportion of the Roma population, increases mortality rates. These results align with earlier analyses of regional differences in perinatal and infant mortality rates that showed similar outcomes [23]. Empirical data from the OECD and the World Bank for 26 OECD countries identifying patterns between education and health indicators, including infant mortality, suggests that with increased levels of education, mortality rates are decreasing [24]. Based on the World Bank data from 127 countries regarding unemployment, with increasing unemployment rates, neonatal and infant mortality is also increasing [25]. Being cautious of generalisation, low educational levels and high unemployment rates are prevalent in the segment of the Roma population living in marginalised Roma communities [26]. On top of the unfavourable socioeconomic conditions of Roma living in social exclusion, many other interconnected factors might contribute to higher mortality rates in Roma, such as malnutrition, maternal smoking during pregnancy, poor hygienic conditions, high prevalence of infectious diseases [27] or worse access and quality of healthcare [28–30]. Thus, our results point out that low education, unemployment and the proportion of disadvantaged Roma population all contribute to the mortality rates of children in Slovakia.

The results from the linear regression models showed that the most significant indicator having a negative impact on mortality in Slovak children is the proportion of the Roma population. This is especially relevant for the period encompassing the COVID-19 pandemic (2020–2022), during which the proportion of the Roma population influenced the neonatal and infant mortality rates the most. Based on the results of a meta-analysis conducted to determine age-specific infection fatality rates for COVID-19, which were very low for children [31], we can assume that other factors related to this period contributed to our results. Shapira, de Walque, and Friedman [32] suggested that several mechanisms related to the economic crisis induced by the COVID-19 pandemic contribute to increased infant mortality rates, which can be observed at the household level and the level of health system disruption. Apart from facing the pandemic, economic downturns were associated with increased mortality of children, mainly in low and middle-income countries but also in high-income countries [33]. Socioeconomic impacts of the COVID-19 pandemic and policy responses such as emergency lockdowns had an unequal impact on ethnic minorities encompassing those in vulnerable situations, including Roma, due to excess poverty, unemployment and social exclusion leading to loss of income, food scarcity, housing insecurity, but also to reduced ability to access already due to pandemic overloaded health services [28, 32, 34]. COVID-19 mobility restrictions, socioeconomic impacts and new forms of discrimination have disproportionately impacted the Roma ethnic minority in many European countries [35]. In Slovakia, whole settlements were quarantined based on the Resolution of the Government of the Slovak Republic [36].

We also found regional differences in the mortality rates, with a higher incidence in the east of Slovakia. One of the factors influencing these regional disparities is the lack of medium- and long-term strategies for economic and regional development and the lack of financial and institutional instruments of regional policy [37]. Another critical factor is the macro-localisation attractiveness, known as the West–East gradient, meaning that regions closer to Western Europe have had better economic and social development conditions than regions in the East, and most undeveloped regions copy the country's eastern borders [38]. Noteworthy is also the structure and character of the settlement network, i.e. the urbanisation of the territory. The regions where large cities are located or where such centres are evenly distributed in the territory are more developed with greater accessibility and quality of service, which significantly impacts the population's quality of life as well as mortality rates and patterns [39]. The absence of a sufficient number of large centres in the regions promotes the widening of regional disparities, which is typical for the less developed regions of Slovakia.

The results indicate that the majority of Roma living in settlements are located in the south and east of Slovakia. The disproportionate spatial distribution of the Roma population in Slovakia is influenced by the history of migration and socioeconomic factors. Southern territories of Slovakia were gradually settled by Roma from the Balkans during the twelfth and thirteenth centuries, followed by the progressive extension of the Roma population in the 15th – 17th to territories with favourable conditions that enabled Roma communities to thrive in crafts, trade, and seasonal work. Over time, municipal and administrative entities began regulating Roma immigration, primarily focusing on economic considerations. By the late 1950s, state authorities in Slovakia had implemented measures to prevent the nomadic lifestyle among the Roma population. Historical developments resulted in Roma communities being represented more prominently in southern and eastern Slovakia, while their presence is relatively lower in northwestern and western regions [40].

### Strengths and limitations

Our study brings insight into socio-economic inequalities in child mortality, focusing on a lower geographical level, which is less common. To determine the public health impact of the explored association, the ecological design, which relies on the availability of data, was used [41]. In ecological study design, measures are based on the average in the population. Thus, caution is needed when applying grouped results to the individual level. Data used in the analyses has also some limitations that need to be mentioned. Atlas of Roma communities comprises settlements or communities with more than 30 residents living concentrated in one place or municipalities with more than 30% share of Roma in the total population [11]. Thus, it does not include Roma living integrated within the majority population in Slovakia. However, Roma living in marginalised Roma communities, as comprised in the Atlas of Roma communities, are disproportionately exposed to health-endangering factors arising from the living environment and socioeconomic conditions and are more vulnerable than those living integrated, which is a more critical aspect from the health perspective than Roma identity as an indicator of socioeconomic deprivation [42].

### Implications

Our results point to the disadvantage of people living in marginalised Roma communities on the top of socioeconomic inequalities. The Strategy of equality, inclusion and participation of the Roma until the year 2030 [43] mentions mortality in children only marginally. Even though the Action plans for this Strategy for the years 2022–2024 aimed to increase life expectancy by reducing neonatal/infant mortality, the proposed measures were insufficient and did not consider the widening gap caused by economic downturn caused by the COVID-19 pandemic. A former global economic analysis, which followed the effect of economic downturns on child mortality for almost 30 years, showed that each child mortality measure continued to display significant deterioration even for up to 5 years after downturns ended and that those at the margins of poverty are less resilient to major social disruptions [33]. Thus, the trend of increased mortality in regions with a higher proportion of the Roma population presumably associated with the economic downturn caused by the COVID-19 pandemic might continue. Thus, concentrated efforts at multiple levels within health and social policies with carefully selected indicators and monitoring processes are needed to reverse this trend. At the community level, continued provision of essential health services to women and children, health mediation services, and supporting programs targeting pregnant women and mothers with children during the most vulnerable pre- and post-natal period is recommended. Future research might focus on factors that can be influenced by policies and evaluation of measures implemented to decrease inequalities in child mortality.

## Conclusion

Socioeconomic disadvantage (proportion of people with only elementary education, long-term unemployment, and proportion of Roma population) negatively affects perinatal, neonatal, and infant mortality rates. The proportion of the Roma population seems to have the most significant impact on mortality in children. The COVID-19 pandemic likely had an unequal impact on the most vulnerable Roma children. Persistent inequities between Roma and the majority population in Slovakia exposed by mortality rates in children point to the vulnerabilities and exposures which were not adequately addressed by health and social policies before the COVID-19 pandemic and were likely even deepened by the policies adopted during the COVID-19 pandemic.

## Data Availability

Datasets generated and/or analysed during the current study are available from the corresponding author on reasonable request.
